# Structural Influence on the Dominance of Virus-Specific CD4 T Cell Epitopes in Zika Virus Infection

**DOI:** 10.3389/fimmu.2018.01196

**Published:** 2018-05-30

**Authors:** Maximilian Koblischke, Karin Stiasny, Stephan W. Aberle, Stefan Malafa, Georgios Tsouchnikas, Julia Schwaiger, Michael Kundi, Franz X. Heinz, Judith H. Aberle

**Affiliations:** ^1^Center for Virology, Medical University of Vienna, Vienna, Austria; ^2^Center for Public Health, Medical University of Vienna, Vienna, Austria

**Keywords:** Zika virus, flavivirus, CD4 T cell, immunodominance, epitope, Zika patients

## Abstract

Zika virus (ZIKV) has recently caused explosive outbreaks in Pacific islands, South- and Central America. Like with other flaviviruses, protective immunity is strongly dependent on potently neutralizing antibodies (Abs) directed against the viral envelope protein E. Such Ab formation is promoted by CD4 T cells through direct interaction with B cells that present epitopes derived from E or other structural proteins of the virus. Here, we examined the extent and epitope dominance of CD4 T cell responses to capsid (C) and envelope proteins in Zika patients. All patients developed ZIKV-specific CD4 T cell responses, with substantial contributions of C and E. In both proteins, immunodominant epitopes clustered at sites that are structurally conserved among flaviviruses but have highly variable sequences, suggesting a strong impact of protein structural features on immunodominant CD4 T cell responses. Our data are particularly relevant for designing flavivirus vaccines and their evaluation in T cell assays and provide insights into the importance of viral protein structure for epitope selection and antigenicity.

## Introduction

Zika virus (ZIKV) has recently caused explosive outbreaks across the Caribbean and the Americas (1). It belongs to the flavivirus genus of the *Flaviviridae* family, along with dengue (DEN), yellow fever (YF), Japanese encephalitis (JE), West Nile (WN), and tick-borne encephalitis (TBE) viruses (2). The severe pathologies associated with ZIKV infection, in particular microcephaly and congenital malformations (3), and its transmission through sexual, congenital, and perinatal routes make it unique among human-pathogenic flaviviruses and have created an urgent need to develop a safe and effective vaccine (4).

Like all flaviviruses, ZIKV is a small (50 nm in diameter) enveloped RNA virus that in its mature form, is composed of multiple copies of three structural proteins, capsid (C), membrane (M), and envelope (E). Immature virions contain prM, the precursor of the M protein, which is proteolytically cleaved during the exocytosis of viral particles (5). The E protein consists of three distinct domains (DI–DIII), followed by a so-called stem region and a double transmembrane anchor. In the mature virus particles, the E protein is arranged in 90 dimers that cover the surface in a specific herringbone-like arrangement (6). Upon entry into host cells, the E protein undergoes an irreversible structural change at the acidic pH of the endosome that converts the E protein from a prefusion dimer into a more stable homotrimer, driving viral membrane fusion (7). Due to its functions in cell entry, the E protein is the principal target of neutralizing antibodies (Abs), and their induction is therefore a primary goal of flavivirus vaccine development (8).

CD4 T cells play an important role in generating such Abs by promoting affinity maturation and the development of B cell memory (9). This helper function is provided by CD4 T cells through direct interaction with B cells, made possible by T cell receptor (TCR) recognition of MHCII-associated peptides presented by the B cells. Direct help can therefore be provided only by peptides derived from proteins that have been internalized by the B cells after recognition through the cognate B cell receptor. In the case of E-specific B cells, such epitopes can be derived from the E protein that is bound by the B cell receptor but also from the other structural proteins, which are internalized as part of the virus particle. Direct CD4 T cell help for the production of neutralizing Abs is therefore restricted to CD4 T cells specific for epitopes derived from the viral structural proteins, whereas this specific function cannot be provided by CD4 T cells specific for epitopes of non-structural proteins (NS proteins). Nevertheless, CD4 T cell responses to NS proteins can provide functions different from direct T-/B cell interactions that may be relevant for controlling ZIKV replication (10).

The activation of CD4 T cells requires their interaction with antigen-presenting cells such as dendritic cells or B cells that present MHCII-associated peptides after proteolytic processing of internalized proteins. In general, only a few selected peptides contained in virus proteins induce such T cell responses, but the mechanisms driving the resulting phenomenon of immunodominance are incompletely understood. Potential factors contributing to the dominance of specific epitopes include the efficacy of their generation during antigen processing, the strength of peptide–MHCII binding and variations in the TCR repertoire (11, 12).

Recent data from cryo-electron microscopy and X-ray crystallography studies showed that the E protein of ZIKV (6, 13, 14) is structurally highly similar to that of other flaviviruses (15), while amino acid sequences differ by up to 60% between distantly related flaviviruses. Because of this combination of structural conservation and sequence divergence, flaviviruses provide excellent models for investigating the roles of protein structure as opposed to sequence in the selection of peptides and the dominance of certain epitopes in CD4 T cell responses.

In this study, we provide the first analysis of the specificities of CD4 T cell responses in Zika patients in the context of the structure of the ZIKV E protein and the two other proteins contained in the virion. Our data show that both C and E contribute substantially to the CD4 T cell response, which was dominated by a few epitopes within each protein. Together with previous studies using YF and TBE viruses (16, 17), a picture emerges that highlights the importance of protein structural features in the selection of peptides and their immunodominance in CD4 T cell responses.

## Materials and Methods

### Study Design

The study took place in Vienna, Austria. For T cell analysis, 14 subjects (8f, 6m; age range, 19–68 years) were recruited at a mean of 10 ± 2 weeks after diagnosis with acute symptomatic ZIKV infection following return to Austria from ZIKV-endemic areas. At the time of diagnosis, 11/14 (79%) patients had detectable ZIKV IgM, one had a borderline Zika IgM-positive result, and 3 patients (Z04, Z05, and Z11) had detectable ZIKV RNA in serum samples. ZIKV infection was confirmed in all cases by virus neutralization assays (Table 1). Patient Z05 was a pregnant woman who developed symptomatic ZIKV infection in the fifth week of her pregnancy and had prolonged viremia for 5 weeks after symptom onset. At the time point of Zika diagnosis, all (14/14) patients were DEN virus IgM negative as well as DEN virus NS1 negative using Denv Detect™ capture ELISA (InBios, Seattle, WA, USA) and Dengue NS1 Detect™ Rapid Test (InBios, Seattle, WA, USA), respectively. Previous DEN virus infection was ruled out using depletion of cross-reactive Abs and post-depletion immunoassays, as described below. Previous TBE or YF vaccination or both TBE and YF vaccinations were confirmed in six (43%), two (14%), and five (36%) subjects, respectively, using neutralization assays as described below. As a control, samples obtained from 10 flavivirus-naive individuals (6f, 4m; age range, 22–47 years) were analyzed.

**Table 1:** Characteristics of study participants.

Patient	Sex/age (years)	Diagnosis of ZIKV infection		Follow-up and CD4 T cell assay						
		ZIKV IgM	ZIKV RNA	Week^a^	ZIKV RNA	ZIKV NT^b^	IL-2 ELISPOT results			
							C	prM	E	Sum
Z01	f/30	pos	nd	4.7	neg	640	81	24	76	181
Z02	f/28	pos	neg	4.7	neg	640	44	1	91	136
Z03	f/41	pos	neg	6.3	neg	320	64	14	61	139
Z04	m/61	neg	pos	9.1	neg	60	30	2	85	117
Z05	f/26	pos	pos	3.3	pos	1,280	27	6	29	62
Z06	f/33	pos	neg	8.0	neg	480	61	8	93	162
Z07	m/21	bdl^c^	neg	27.3	neg	320	78	0	73	151
Z08	f/43	pos	neg	10.1	neg	120	74	30	76	180
Z09	f/19	pos	neg	5.4	neg	120	27	20	69	116
Z10	m/25	pos	neg	5.4	neg	320	64	34	104	202
Z11	m/29	neg	pos	26.0	neg	120	33	5	28	66
Z12	f/38	pos	neg	8.4	neg	160	57	2	77	136
Z14	m/68	pos	neg	13.0	neg	960	102	36	139	277
Z15	m/67	pos	neg	8.4	neg	640	73	12	43	128

*^a^Week from onset of symptoms*.

*^b^ZIKV neutralization titer*.

*^c^Borderline positive result*.

*ZIKV, Zika virus*.

### Ethics Statement

This study was carried out in accordance with the recommendations of the Declaration of Helsinki. The protocol was approved by the ethics committee of the Medical University of Vienna, Austria (approval no. 1295/2016). All subjects gave written informed consent.

### Preparation of Peripheral Blood Samples

Peripheral blood mononuclear cells (PBMCs) were isolated from whole-blood samples using Ficoll-Paque Plus™ (GE Healthcare) and cryopreserved in liquid nitrogen. PBMCs were depleted of CD8 cells using anti-CD8 Ab-coupled magnetic beads and LD columns (Miltenyi Biotec GmbH, Germany), as previously described (17). CD8-depleted PBMCs were incubated overnight at 37°C in 5% CO_2_ in serum-free medium (AIM-V; Gibco) and resuspended at a final concentration of 2 × 10^6^ cells/ml in AIM-V. The purity of CD8-depleted PBMCs in each sample was assessed by flow cytometry using anti-CD8-APC, anti-CD3-PE, anti-CD4-PacificBlue™, and 7-aminoactinomycin D (all purchased from BD Bioscience), which showed that purity of CD8-depleted PBMCs was >99%.

### Peptides

A total of 192 15-mer peptides overlapping by 11 amino acids were purchased from JPT (Berlin, Germany). The peptides cover the entire sequences of C, prM, and E from the ZIKV strain H/PF/2013 (protein accession code: AHZ13508). The purity of peptides was >70%, as determined by high-performance liquid chromatography. Lyophilized peptides were dissolved in dimethyl sulfoxide and diluted in AIM-V. The peptides were grouped into three master pools to cover each of the C, prM, and E protein sequences. Matrix pools of C (*n* = 10) and E (*n* = 22) contained up to 14 peptides with each peptide present in two different pools. All positive results obtained with the matrix pools were confirmed by testing the samples with single peptides.

### IL-2 ELISPOT Assay

The IL-2 ELISPOT assay was performed as described previously (17). Briefly, plates (Merck-Millipore) were coated with anti-IL-2 Ab (3445-3-1000, Mabtech) and blocked with RPMI 1640 (Sigma) containing 10% human serum, 1% penicillin/streptomycin/glutamine (Gibco), and 1% non-essential amino acids (Sigma). 2 × 10^5^ CD8-depleted PBMCs were added per well. The cells were incubated for about 45 h at 37°C and 5% CO_2_ with either peptides at a final concentration of 2 µg/ml or AIM-V (negative control) or phytohemagglutinin (PHA, Sigma) at a final concentration of 0.5 µg/ml (positive control). After washing, spots were developed with biotin-conjugated Ab (3445-6-250, Mabtech), streptavidin alkaline phosphatase (ALP; 1:1,000, 3310-10, Mabtech), and 5-bromo-4-chloro-3-indolylphosphate/nitroblue tetrazolium (BCIP/NBT; B5655, Sigma). The dried plates were analyzed using a Bio-Sys Bioreader 5000 Pro-S/BR177, generation 10. Data were calculated as spots per 1 × 10^6^ CD8-depleted PBMCs after subtraction of the negative control (mean spot number from three to four unstimulated wells), as described previously (17). The response to a single peptide was defined positive if the corresponding master pool, matrix pool, and single-peptide testing yielded >20 spots per 1 × 10^6^ CD8-depleted PBMCs.

### Structural Analysis

Zika virus epitopes identified in this study were assigned to the crystallographic structures of ZIKV sE (PDB 5LBV) and KUNV C (PDB 1SFK) as well as to the cryo-EM structure of ZIKV E, which includes the stem and transmembrane regions (PDB 5IZ7). For comparing the epitopes from different flaviviruses, we used data from the IEDB that were derived from *ex vivo* ELISPOT assays with human CD4 T cells and overlapping peptides that covered the entire protein sequence (16–19). To further evaluate the significance of the immunodominant regions identified, we performed an analysis of all published, experimentally determined HLA class II-restricted flavivirus epitopes from the IEDB. In total, 1,225 additional epitope data were retrieved. These included 218 peptide entries derived from DEN virus C or E proteins that were positive in at least two responders (20, 21). Epitopes from DEN (18, 19), YF (16), and TBE (17) viruses were assigned to the structures of KUN virus C (PDB 1SFK), DEN-2 sE (PDB 1OAN), and TBE sE (PDB 1SVB), respectively. For epitope assignment onto heterologous protein structures, multiple sequence alignments were performed (GenBank: ZIKV KJ776791; DEN 1–4 viruses AF226687, M29095, DQ863638, GQ398256; YF virus CAA27332; WN virus DQ211652; JE virus D90194 and TBE virus U27495) using the Clustal Omega online tool (22). Secondary structures of ZIKV C protein were predicted by use of PSIPRED algorithm.^1^

### Analysis of Protein Sequence Identity

Amino acid sequences of mature C and E proteins from ZIKV (GenBank KJ776791), DEN 1–4 (GenBank AF226687, M29095, DQ863638, GQ398256), YF (GenBank CAA27332), WN (GenBank DQ211652), JE (GenBank D90194), and TBE (GenBank U27495) viruses were aligned using Clustal Omega and manually refined as described previously (15, 23).

### ZIKV Nucleic Acid Amplification Test

Nucleic acid extraction was performed in an automated manner according to the manufacturer’s instructions (NucliSENS^®^ easyMAG^®^, bioMérieux, France). ZIKV-specific real-time TaqMan PCR was performed with primers and probe located in the NS5 gene, as described (24).

### Ab Depletion

Previous DEN virus infection was ruled out using depletion of cross-reactive Abs and DEN-IgG ELISA assays (Anti-Dengue Virus ELISA IgG, EUROIMMUN, Lübeck, Germany). Depletion of broadly cross-reactive Abs from sera was performed using Strep-Tactin spin columns (IBA GmbH, Göttingen, Germany). Recombinant WN virus soluble E protein with a C-terminal Strep-Tag (25) was bound to Strep-Tactin XT Spin Columns according to the manufacturers’ instructions. Serum samples were diluted 1:10 in PBS, pH 7.4, 0.1% BSA and loaded onto the columns following the protocol of the manufacturer. After centrifugation, the flow-through was collected, reapplied 6 times and quantified in a DEN-IgG ELISA. The data obtained were expressed as the percentage of the values (relative units) of the non-depleted sera. Due to the limited amount of serum available, these experiments were carried out with samples from 12 of the 14 Zika patients. DEN-IgG reactivity was below the detection limit in nine patients indicating that they were DEN-naïve (Z03, Z04, Z07, Z09, Z10, Z11, Z12, Z14, and Z15). As expected patient Z05 had residual DEN-IgG reactivity (10.4% of the non-depleted serum). In the depleted serum of patient Z08, 5.4% of the DEN-IgG Abs was not removed by the heterologous E protein, and patient Z02 had very low residual DEN-IgG reactivity after depletion (<1% of the non-depleted serum).

### Neutralization Assays

Serial twofold dilutions of heat-inactivated serum samples (duplicates) were mixed with 30–60 TCID_50_ ZIKV (strain H/PF 2013, European Virus Archive^2^) and incubated for 1 h at 37°C. Vero cells (ECACC) were added and incubation was continued for 3–4 days. Virus neutralization titers were expressed as the reciprocal of the serum dilution that was required for 100% protection against virus-induced cytopathic effects. For YF virus neutralization assays, heat-inactivated serum samples were incubated with 40–80 TCID_50_/well of YF virus for 1 h at 37°C. The presence of virus in the supernatant was assessed by the occurrence of cytopathic effects. Virus neutralization titers ≥20 were considered positive.

Tick-borne encephalitis virus neutralization assays were carried out as described previously (26). In brief, serial dilutions of plasma samples (in duplicates) were mixed with 25 PFU of TBE virus strain Neudörfl and incubated for 1 h at 37°C. Baby hamster kidney cells (ATCC BHK-21) were added, and incubation was continued for 3 days. The presence of virus in the supernatant was assessed by ELISA. The virus neutralization titer was defined as the reciprocal of the plasma sample dilution that gave a 90% reduction in the absorbance readout in the assay compared with the control without Ab. Virus neutralization titers ≥10 were considered positive.

### Statistical Analysis

A general linear model was applied to estimate the impact of a previous YF and/or TBE vaccination on the extent and breadth of ZIKV-specific CD4 T cell responses. With a log-link model that was tested for normality of residuals and homogeneity of variances by Kolmogorov–Smirnov (Lilliefors correction) and Levene’s tests, respectively, deviations from the assumption of no influence were tested at the *p* = 0.05 level of significance. The fractions of conserved amino acids in the common epitope regions and non-dominant epitope regions of C and E were compared using exact tests based on the hypergeometric distribution. The probability of overlap between ZIKV epitopes and those of other flaviviruses was tested separately for C and E by binomial tests.

## Results

### CD4 T Cell Responses to ZIKV Structural Proteins C, prM, and E

**Figure 1:**
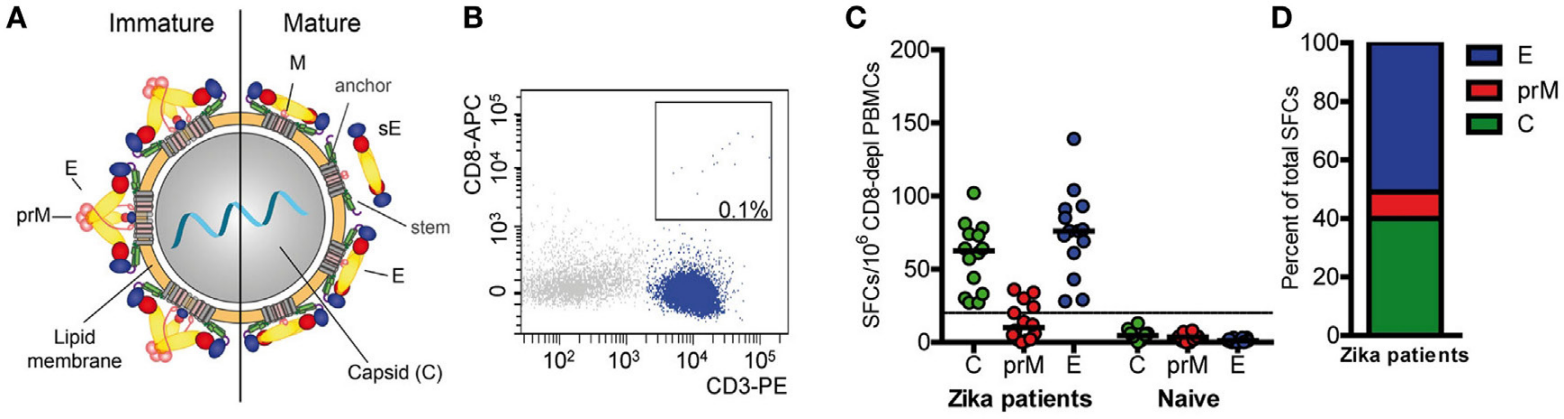
CD4 T cell responses to Zika virus infection. **(A)** Schematics of a flavivirus particle representing an immature (left) and mature (right) virion that comprises three structural proteins: C (capsid), prM (membrane), and E (envelope). **(B)** Representative FACS plot from CD8-depleted peripheral blood mononuclear cells (PBMCs). **(C)** Individual CD4 T cell responses to C, prM, and E from Zika patients and naïve individuals as determined in IL-2 ELISPOT assays. Results are given as spot-forming cells (SFCs). Medians are depicted as black lines. The dashed line represents the cutoff for assay positivity. **(D)** Percentage of spots contributed by C, prM, and E peptides in Zika patients.

Overall CD4 T cell responses to the ZIKV structural proteins were determined by IL-2 ELISPOT assays using PBMCs from 14 patients with laboratory-confirmed ZIKV infection and peptide pools of C, prM, and E (Figure 1A). To specifically determine CD4 T cell responses, PBMC was depleted of CD8 cells before stimulation with ZIKV peptides. Flow cytometry confirmed that purity of CD8-negative PBMCs was >99% (Figure 1B). These analyses were carried out at a mean (±SEM) of 10 ± 2 weeks after onset of clinical symptoms (Table 1), and the results are presented in Figure 1C.

CD4 T cells specific for C and E proteins were detectable in 100% of patients and in 36% for prM, but in none of the flavivirus-naïve controls. Fifty percent of the total response was accounted by E peptides, 40% by C peptides and 10% by prM peptides (Figure 1D). Under the assumption that epitopes from C and E would equally contribute to the response, the C response was approximately twofold higher than expected from its molecular weight. A similar overrepresentation of the response to C has also been found after TBE virus infection and vaccination as well as after YF vaccination (16, 17, 27).

**Figure 2:**
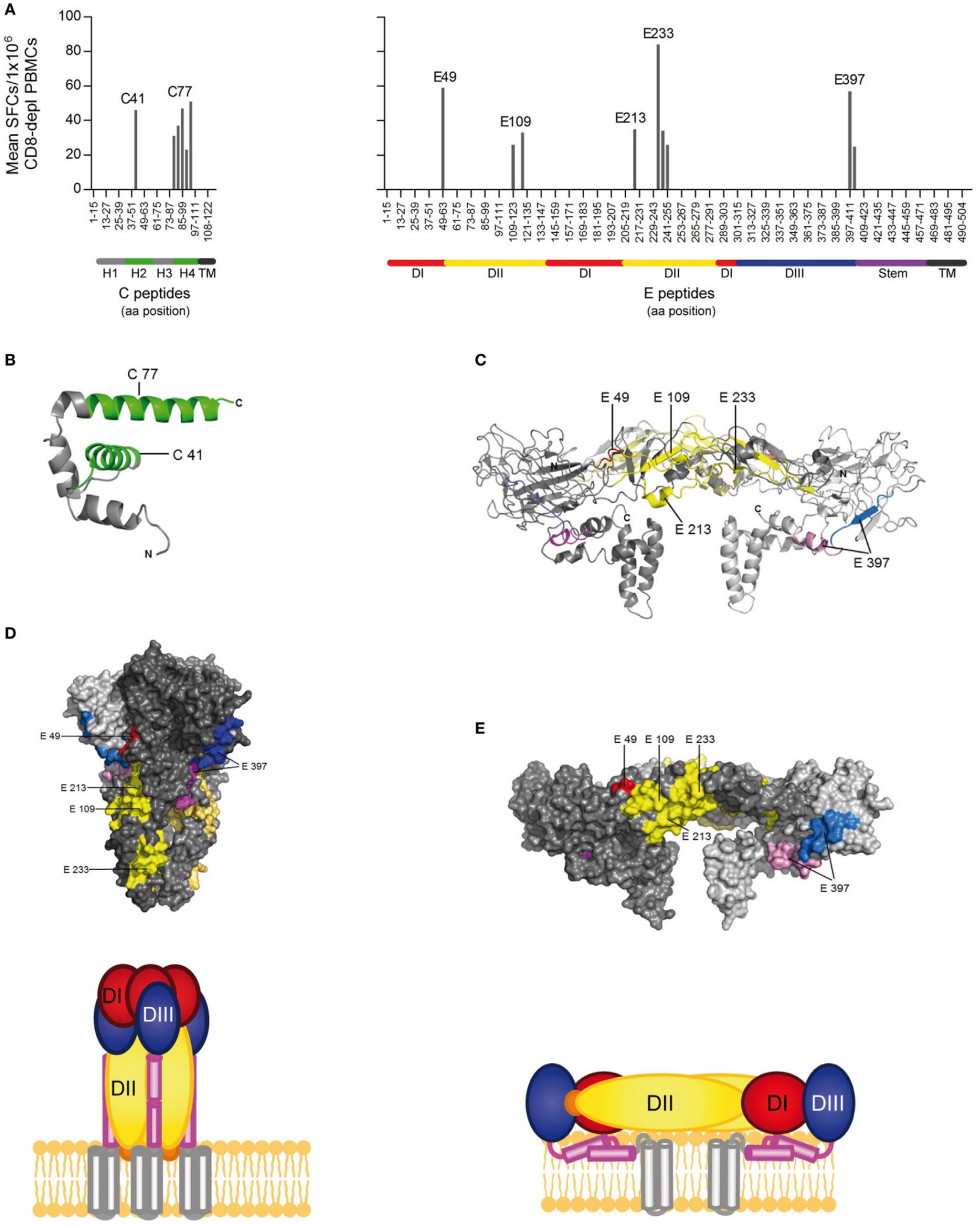
CD4 T cell epitope mapping for Zika virus (ZIKV) C and E proteins. **(A)** Mean spot count of immunodominant responses to single peptides detected in ≥20% of Zika patients. Amino acid positions of peptides in C and E protein sequences are indicated on the *x*-axes. Epitope clusters are denoted by the first amino acid of the N-terminal 15-mer peptide used for single-peptide testing. **(B)** Ribbon representation of the flavivirus Kunjin (KUN) C protein (PDB 1SFK) (28), comprising four helices. **(C)** Ribbon representation of the ZIKV E dimer (PDB 5IZ7; side view) (6), consisting of the three domains (DI–III), the stem, and transmembrane anchor. ZIKV epitopes recognized by ≥20% of Zika patients are colored as follows: C—green; E: domain I—red; domain II—yellow; domain III—blue; stem—purple, transmembrane domain (TM)—black. **(D)** Surface representation of the truncated dengue (DEN)-1 virus postfusion trimeric form of E and a schematic showing a model of the full-length trimer (DEN-1; PDB 4GT0; side view) (29). **(E)** Surface representation of the ZIKV E dimer (PDB 5IZ7; side view) and the corresponding schematic.

To determine the epitope specificities of CD4 T cell responses, we performed IL-2 ELISPOT assays with peptide matrix pools (Figure S1 in Supplementary Material) as well as single peptides from C and E with CD8-depleted PBMCs from all 14 patients. In two instances, the number of PBMCs available allowed testing of either only C or E peptides, thus generating sets of single C and E peptide responses for 13 patients. The complete list of epitopes identified in C and E proteins is displayed in Tables 2 and 3, together with the percentage of responders out of all individuals reacting with at least one peptide in C or E. Of the 54 peptides identified, 15 were recognized by ≥20% of patients and were thus the most dominant targets of ZIKV CD4 T cell responses (Figure 2A). The same pattern was obtained when the evaluation was based not only on the most dominant targets but on all peptide responses (Figure S2 in Supplementary Material). Each individual recognized 1–5 of these 15 dominant epitopes, 6 of which were derived from C and 9 from E. One patient (Z05) had no detectable single-peptide response, consistent with the low overall Zika CD4 T cell response in this individual (Table 1).

**Table 2:** Identified CD4 T cell epitopes from Zika virus C protein.

Peptide position^a^	Peptide sequence	Responders (%)^b^
21–35	VARVSPFGGLKRLPA	10
25–39	SPFGGLKRLPAGLLL	10
29–43	GLKRLPAGLLLGHGP	10
33–47	LPAGLLLGHGPIRMV	10
37–51	LLLGHGPIRMVLAIL	10
**41–55**	**HGPIRMVLAILAFLR**	**20**
61–75	PSLGLINRWGSVGKK	10
65–79	LINRWGSVGKKEAME	10
73–87	GKKEAMEIIKKFKKD	10
**77–91**	**AMEIIKKFKKDLAAM**	**20**
**81–95**	**IKKFKKDLAAMLRII**	**30**
**85–99**	**KKDLAAMLRIINARK**	**30**
**89–103**	**AAMLRIINARKEKKR**	**20**
**93–107**	**RIINARKEKKRRGAD**	**30**
101–115	KKRRGADTSVGIVGL	10

*^a^Amino acid position within the C protein*.

*^b^Percentage of responders recognizing a specific single peptide*.

*Peptides recognized in ≥20% of responders are indicated in bold*.

**Table 3:** Identified CD4 T cell epitopes from Zika virus E protein.

Peptide position^a^	Peptide sequence	Responders (%)^b^
**49–63**	**TVSNMAEVRSYCYEA**	**20**
53–67	MAEVRSYCYEASISD	10
81–95	YLDKQSDTQYVCKRT	10
89–103	QYVCKRTLVDRGWGN	10
105–119	CGLFGKGSLVTCAKF	10
**109–123**	**GKGSLVTCAKFACSK**	**20**
113–127	LVTCAKFACSKKMTG	10
**117–131**	**AKFACSKKMTGKSIQ**	**30**
121–135	CSKKMTGKSIQPENL	10
133–147	ENLEYRIMLSVHGSQ	10
145–159	GSQHSGMIVNDTGHE	10
149–163	SGMIVNDTGHETDEN	10
153–167	VNDTGHETDENRAKV	10
157–171	GHETDENRAKVEITP	10
165–179	AKVEITPNSPRAEAT	10
173–187	SPRAEATLGGFGSLG	10
209–223	KHWLVHKEWFHDIPL	10
**213–227**	**VHKEWFHDIPLPWHA**	**20**
229–243	ADTGTPHWNNKEALV	10
**233–247**	**TPHWNNKEALVEFKD**	**20**
**237–251**	**NNKEALVEFKDAHAK**	**20**
**241–255**	**ALVEFKDAHAKRQTV**	**20**
245–259	FKDAHAKRQTVVVLG	10
249–263	HAKRQTVVVLGSQEG	10
309–323	TAAFTFTKIPAETLH	10
321–335	TLHGTVTVEVQYAGT	10
329–343	EVQYAGTDGPCKVPA	10
333–347	AGTDGPCKVPAQMAV	10
337–351	GPCKVPAQMAVDMQT	10
341–355	VPAQMAVDMQTLTPV	10
365–379	ITESTENSKMMLELD	10
373–387	KMMLELDPPFGDSYI	10
381–395	PFGDSYIVIGVGEKK	10
**397–411**	**THHWHRSGSTIGKAF**	**30**
**401–415**	**HRSGSTIGKAFEATV**	**20**
409–423	KAFEATVRGAKRMAV	10
441–455	LGKGIHQIFGAAFKS	10
457–471	FGGMSWFSQILIGTL	10
465–479	QILIGTLLMWLGLNT	10

*^a^Amino acid position within the E protein*.

*^b^Percentage of responders recognizing a specific single peptide*.

*Peptides recognized in ≥20% of responders are indicated in bold*.

### Structural Analysis of Epitope Sites

Because the structural context of epitopes has been proposed to influence immunodominance (30), we analyzed the location of dominant epitopes in the structure of the ZIKV E protein (6, 13) and the C protein from WN/Kunjin virus (28). This is so far the only available atomic structure of a flavivirus C protein, which, however, is considered to have a similar overall conformation in ZIKV, based on the structural conservation of flavivirus proteins in general. For C, epitopes (designated *C41* and *C77*) were identified in helices 2 and 4 of the protein, respectively (Figure 2B). In E, dominant epitopes clustered in each of the three domains [one in domain I (*E49*), three in domain II (*E109, E213* and *E233*), one in domain III (*E397*), and the adjacent stem region (*E401*)] (Figure 2C). As shown in the protein surface representation of the E dimer (Figure 2E), these epitopes are exposed at the outside of the molecule. They also have an external location in the trimeric, low-pH conformation of E, which is acquired upon viral membrane fusion in the endosome (Figure 2D).

**Figure 3:**
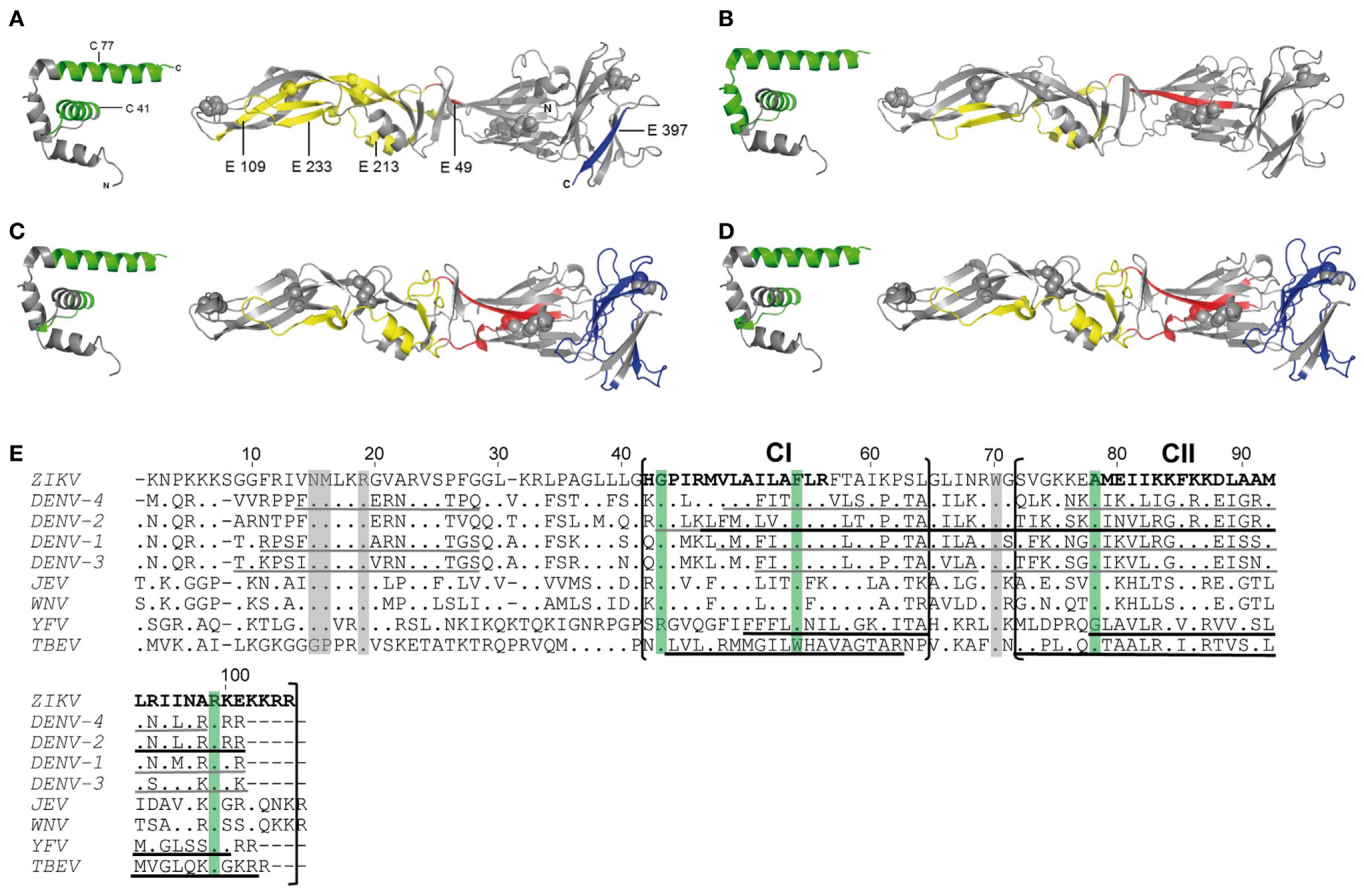

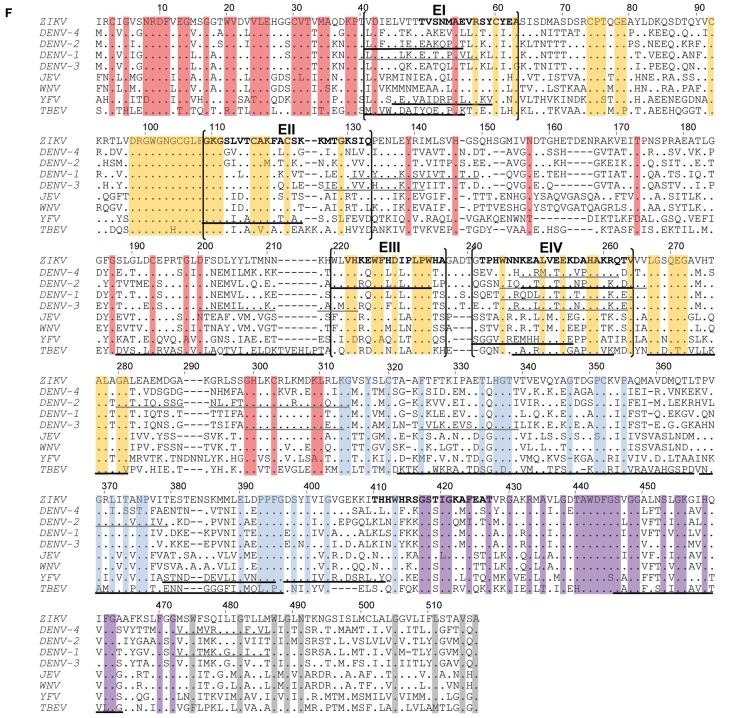
Comparison of CD4 T cell epitope regions in C and E proteins of flaviviruses. [**(A–D)**, left panel] Ribbon representation of the flavivirus Kunjin (KUN) C protein (PDB 1SFK). [**(A–D)**, right panel] Ribbon representations of Zika virus (ZIKV) sE (PDB 5LBV) (13) **(A)**, dengue (DEN)-2 virus sE (PDB 1OAN) (31) **(B)**, tick-borne encephalitis (TBE) virus sE (PDB 1SVB) viruses (32) **(C,D)**. Epitopes from Zika **(A)**, DEN-2/4 (18, 19) **(B)**, yellow fever (YF) (16) **(C)**, and TBE (17) **(D)** viruses are colored as follows: C—green; E: domain I—red; domain II—yellow; domain III—blue. **(E,F)** Capsid **(E)** and envelope **(F)** protein sequence alignments of nine human-pathogenic flaviviruses. The C and E protein sequences from Zika (GenBank KJ776791), dengue (DEN) 1–4 (GenBank AF226687, M29095, DQ863638, GQ398256), Japanese encephalitis (JE) (GenBank D90194), West Nile (WN) (GenBank DQ211652), YF (GenBank CAA27332), and TBE (GenBank U27495) viruses were aligned using CLUSTAL omega. Experimentally determined epitopes from ZIKV are indicated in bold, those from DEN (18, 19), YF (16), and TBE (17) viruses are underlined in black and additional epitopes retrieved from the IEDB in gray. Sequence elements with ≥90% amino acid identity across all flaviviruses are highlighted as follows: C—green; E: domain I—red; domain II—yellow; domain III—blue; stem—purple; TM—gray.

Comparison of the newly identified immunodominant ZIKV epitopes with those identified for YF (16), DEN (18, 19), and TBE (17) viruses revealed remarkable similarities for both C and E epitopes for all four flaviviruses (Figures 3A–D). Immunodominant epitope regions that are shared between flaviviruses are shown in Figures 3E,F and are designated CI, CII, and EI–EIV for C and E proteins, respectively. Statistical analysis revealed a significant overlap of these epitope regions compared with random allocation for both C and E of all four flaviviruses (*p* < 10^−15^, binomial test). The most outstanding similarity was the coincidence of four immunodominant regions in all flaviviruses investigated, two in C (CI and CII, Figure 3E) and two in E (EI and EIV, Figure 3F). In addition, shared epitope regions were found between ZIKV and other mosquito-borne flaviviruses, including one with YF (EII) and one with DEN (EIII), but not with TBE virus. Such a difference was also found with respect to domain III epitopes, which were overrepresented in TBE virus, but not in mosquito-borne flaviviruses (Figure 3F).

As a further evaluation of the significance of the immunodominant regions identified, we performed an analysis of all experimentally determined HLA class II-restricted flavivirus epitopes from the Immune Epitope Database (IEDB). In total, 1,225 additional peptides were retrieved. These data included 218 peptide entries derived from DEN virus C and E proteins identified by *ex vivo* ELISPOT assays in at least two responders (20, 21). Significantly, 86 and 69% of all epitopes mapped to one of the immunodominant regions in C or E identified in our work (*p* < 10^−15^, binomial test) (Figures 3E,F).

To assess whether the similarities of immunodominant epitopes observed among distantly related flaviviruses were due to sequence conservation, the percentage of identical amino acids was calculated for each epitope region in the aligned protein sequences (Figures 3E,F). The analysis clearly showed that these dominant epitopes were poorly conserved among flaviviruses, with only 22 and 23% sequence identities for CI and CII, respectively, and 42% (EI), 52% (EII), 65% (EIII), and 43% (EIV) for epitopes in E. Sequence conservation of dominant epitopes did not differ significantly from non-dominant epitope regions, both in C (*p* = 0.804) and E (*p* = 0.645), as revealed by hypergeometric tests. Similar results were obtained when we used consensus sequences for ZIKV and for DEN viruses 1–4, as reported recently (33), rather than sequences of individual strains as displayed in Figures 3E,F. The data demonstrate that sequence conservation was not responsible for the similarities of immunodominant epitope patterns observed with different flaviviruses. Taken together, the similar distributions of immunodominant CD4 T cell epitopes with divergent sequences in the C and E proteins of different flaviviruses are consistent with an important role of conserved structural features that favor the generation of certain peptides and their immunodominance in CD4 T cell responses.

## Discussion

CD4 T cells specific for epitopes of the flavivirus structural proteins can provide help to B cells by direct cell–cell interactions and are thus essential for the production of high-affinity neutralizing Abs against E. This function makes them key components of a potent and long-lived immunity against flavivirus infections. In this work, we provide the first description of the epitope specificities in such CD4 T cell responses after ZIKV infection in humans and an analysis of the results in the context of the three-dimensional structures of the capsid and envelope proteins.

Zika patients mounted strong CD4 T cell responses to epitopes from both the C and E proteins. Evaluations based on either responder frequencies or the magnitude of responses yielded similar results for C and E peptides. The equivalence of the response to these two structural proteins mirrors the situation observed after YF vaccination as well as after TBE virus infection and vaccination (16, 17). As shown for TBE virus, the number of C molecules in the virion is twofold to threefold higher than that of E, which can provide an explanation for the similar extents of CD4 T cell responses to both proteins, although their molecular weights differ substantially (12 vs 54 kDa). Based on the concept that E-specific B cells can internalize whole virus particles, they can receive help through the interaction with specific CD4 T cells not only through the presentation of peptides derived from E, but also from C. Such intra-particle help mechanisms have been demonstrated in studies with hepatitis B (34) and influenza (35, 36) and were identified as important determinant of improved Ab responses against HIV (37). Considering the enormous potential of chimeric vaccine candidates for the control of highly pathogenic flaviviruses, a better understanding of such intra-particle help mechanisms is of paramount importance for optimizing humoral and T cell responses.

Despite individual-specific variations, the CD4 T cell response in Zika patients was focused to a few peptides that dominated the response. For measuring peptide reactivities, we used an IL-2 ELISPOT assay that was applied in previous studies for YF (16) and TBE (17) viruses and enabled a direct comparison of the newly identified Zika CD4 T cell epitopes with those of other flaviviruses. The present analysis revealed a similar distribution of epitopes between IL-2 and interferon-gamma ELISPOT assays with low responder frequencies to individual epitopes (16–21).

So far, the molecular mechanisms involved in the selection of immunodominant epitopes are not entirely clear, but in addition to host-specific factors can be influenced by protein structural features that affect epitope processing (30). Such features might affect antigen processing and epitope presentation through influencing their availability for MHCII binding and/or proteolytic processing (38). Our study substantiates such structural influences by demonstrating conserved epitope dominance patterns in the CD4 T cell responses after ZIKV and other flavivirus infections or vaccinations. Especially with epitopes of the C protein, a striking similarity was observed between Zika, DEN, YF, and TBE virus-specific CD4 T cells (16–21). They all targeted predominantly two alpha helices in the C proteins (Figures 3A–D) that have highly variable sequences in different flaviviruses (only 22 and 23% identical amino acids), demonstrating that sequence conservation was not responsible for the epitope dominance observed.

For epitopes of the E protein, the situation appears to be more complex. There was remarkable similarity of epitope regions in E domains I and II, where we identified two epitope sites shared in all four flaviviruses and two additional epitope regions that were shared between Zika and other mosquito-borne viruses, i.e., DEN or YF viruses, but not with TBE virus. By contrast, distinct preferences for specific regions were observed with respect to domain III, which was clearly overrepresented in TBE but not in mosquito-borne flaviviruses.

In the case of E, the substrate for generating peptides may not only be its native dimeric conformation present on infectious virions but also the trimeric conformation generated in the course of viral membrane fusion (7). Because the fusogenic conformational change can be triggered already by the mildly acidic pH in early endosomes, the postfusion trimer could serve as epitope source for CD4 T cells. Mapping of immunodominant ZIKV epitopes to the three-dimensional structures of the prefusion E dimer and the postfusion trimer showed that most were accessible at exposed surfaces in both conformations. Of note, epitopes in the stem region of E (such as *E401*) are cryptic in the context of prefusion virus particles but become exposed after membrane fusion. The postfusion conformation may therefore be the primary substrate for generating these epitopes. This hypothesis is supported by previous studies with TBE virus, which showed that stem epitopes are immunodominant only in patients after natural infection but not after vaccination with an inactivated vaccine, in which the E protein is fixed in its prefusion conformation by treatment with formalin (17).

Taken together, the similar distributions of immunodominant CD4 T cell epitopes with divergent sequences in the C and E proteins of different flaviviruses are consistent with an important role of conserved structural features that favor the generation of certain peptides during antigen processing. Evidence for a structural influence also comes from studies of CD4 T cell responses to HIV and influenza proteins, which showed that the corresponding T cell epitopes are located at exposed protein elements or at flanks of conformationally flexible loops (39–41).

Although our results emphasize the importance of protein structure for controlling the immunodominance of CD4 T cell epitopes, additional parameters must be considered that could influence epitope selection. These may include virus-specific factors that antagonize host innate immune responses (42) or the lysosomal protease activity (43). In addition, individual-specific factors, such as differences in the naive TCR repertoire or stability of TCR/peptide–MHC interactions (11) may contribute to the epitope patterns observed. Also, preexisting immunity from previous infection with related flaviviruses could alter immunodominance patterns of CD4 T cells through cross-reactive memory responses to conserved elements. Sequence conservation, however, was not observed for the dominant ZIKV epitopes identified in our work, which exhibited only 22–23% (C peptides) and 42–65% (E peptides) identical amino acids compared with corresponding sequences in other flaviviruses. Furthermore, we did not find any evidence for an impact of a previous YF and/or TBE vaccination on the breadth or dominance of ZIKV epitope responses when we compared the groups with either a YF or a TBE vaccination or both TBE and YF vaccinations (Table 4), although the extents of CD4 T cell reactivities to ZIKV proteins were higher in those with a previous YF vaccination (*F* = 5.77, *p* = 0.03, general linear model). A previous DEN virus infection was ruled out in the majority of Zika patients using depletion of cross-reactive Abs and post-depletion immunoassays. Only one patient (Z05) had substantial DEN-IgG reactivity in the post-depletion serum, confirming previous DEN infection. This patient had low overall ZIKV-specific CD4 T cell responses, and no single-peptide responses were detected. The patient acquired ZIKV infection during early pregnancy and had prolonged viremia for 5 weeks, consistent with a recent study reporting Zika viral RNA persistence in the blood of pregnant women (44).

**Table 4:** Zika virus CD4 T cell responses in patients with previous tick-borne encephalitis (TBE) or yellow fever (YF) vaccination.

Subject group	Magnitude of response^b^ Medians (range)	Breadth of response^c^	
		All peptides Medians (range)	Dominant peptides Medians (range)
**Previous vaccination^a^**			
YF and TBE (*n* = 5)	151 (117–277)	6 (1–18)	2 (1–3)
YF (*n* = 2)	154 (128–180)	5 (4–5)	2
TBE (*n* = 6)	136 (66–202)	6 (1–11)	3 (1–5)

*^a^One patient (Z05) had no detectable single-peptide response and was therefore not included in the comparison*.

*^b^Sum of responses to C, prM, and E peptide pools (SFCs/10^6^ cells)*.

*^c^Number of peptides recognized*.

Our study corroborates that structural features of proteins contribute substantially to the selection of CD4 T cell epitopes and thus have a strong impact on immunodominance. Considering the important role of CD4 T cells in promoting affinity maturation and the development of B cell memory, insights into the mechanisms controlling their specificities are of paramount importance for understanding how potent protective immunities are induced by natural infection or vaccination.

## Data Availability Statement

All datasets generated for this study are included in the manuscript.

## Ethics Statement

This study was carried out in accordance with the recommendations of the declaration of Helsinki. The protocol was approved by the ethics committee of the Medical University of Vienna, Austria. All subjects gave written informed consent in accordance with the Declaration of Helsinki.

## Author Contributions

MK, JA, FH, SA, KS, SM, GT, and JS performed experiments, reviewed data, and planned experimental strategy. MK performed the statistical analysis. FH and JA conceived and directed the study and wrote the manuscript. All the authors critically read and edited the manuscript.

## Conflict of Interest Statement

The authors declare that the research was conducted in the absence of any commercial or financial relationships that could be construed as a potential conflict of interest.

## Footnotes

^1^http://bioinf.cs.ucl.ac.uk/psipred (Accessed: September 2017).

^2^https://www.european-virus-archive.com (Accessed: April 2015).

## Abbreviations

Abs, antibodies; DEN, dengue; JE, Japanese encephalitis; NS protein, non-structural protein; PBMCs, peripheral blood mononuclear cells; TCR, T cell receptor; TBE, tick-borne encephalitis; YF, yellow fever; WN, West Nile.
