# Corrigendum: Structural Influence on the Dominance of Virus-Specific CD4 T Cell Epitopes in Zika Virus Infection

**DOI:** 10.3389/fimmu.2018.02083

**Published:** 2018-09-11

**Authors:** Maximilian Koblischke, Karin Stiasny, Stephan W. Aberle, Stefan Malafa, Georgios Tsouchnikas, Julia Schwaiger, Michael Kundi, Franz X. Heinz, Judith H. Aberle

**Affiliations:** ^1^Center for Virology, Medical University of Vienna, Vienna, Austria; ^2^Center for Public Health, Medical University of Vienna, Vienna, Austria

**Keywords:** Zika virus, flavivirus, CD4 T cell, immunodominance, epitope, Zika patients

An author's name was incorrectly spelled as Georgios Tschouchnikas. The correct spelling is Georgios Tsouchnikas. The authors apologize for this error and state that this does not change the scientific conclusions of the article in any way.

The original article has been updated.

## Conflict of interest statement

The authors declare that the research was conducted in the absence of any commercial or financial relationships that could be construed as a potential conflict of interest.

