# Supplementary material for: Structural Influence on the Dominance of Virus-Specific CD4 T Cell Epitopes in Zika Virus Infection
Source: Front Immunol. 2018 May 30;9:1196. doi: 10.3389/fimmu.2018.01196 (PMC5989350; doi:10.3389/fimmu.2018.01196)
Supplement: Supplementary file 1 [file Data_Sheet_1.PDF]

Supplementary Material

Structural influence on the dominance of virus-specific CD4 T cell epitopes in Zika virus infection

Maximilian Koblischke[1], Karin Stiasny[1], Stephan W. Aberle[1], Stefan Malafa[1], Georgios Tschouchnikas[1], Julia Schwaiger[1], Michael Kundi[2], Franz X. Heinz[1] and Judith H. Aberle[1]*

[1]Center for Virology, Medical University of Vienna, [2]Center for Public Health, Medical University of Vienna, Vienna, Austria.

***Correspondence:** Dr. Judith Aberle: judith.aberle@meduniwien.ac.at

A

Zika virus C

**Pool**	**I**	**II**	**III**	**IV**	**V**
**VI**	1	2	3	4	5
**VII**	6	7	8	9	10
**VIII**	11	12	13	14	15
**IX**	16	17	18	19	20
**X**	21	22	23/26	24/27	25/28

B

Zika virus E

**Pool**	**I**	**II**	**III**	**IV**	**V**	**VI**	**VII**	**VIII**	**IX**	**X**	**XI**
**XII**	1	2	3	4	5	6	7	8	9	10	11
**XIII**	12	13	14	15	16	17	18	19	20	21	22
**XIV**	23	24	25	26	27	28	29	30	31	32	33
**XV**	34	35	36	37	38	39	40	41	42	43	44
**XVI**	45	46	47	48	49	50	51	52	53	54	55
**XVII**	56	57	58	59	60	61	62	63	64	65	66
**XVIII**	67	68	69	70	71	72	73	74	75	76	77
**XIX**	78	79	80	81	82	83	84	85	86	87	88
**XX**	89	90	91	92	93	94	95	96	97	98	99
**XXI**	100	101	102	103	104	105	106	107	108	109	110
**XXII**	111	112	113	114	115	116	117	118	119/122	120/123	121/124

Figure S1. Two-dimensional peptide matrix. The single peptides of Zika virus C **(A)** and E **(B)** proteins were arranged into a matrix with each individual peptide present in two separate pools (Roman numerals). The individual peptides (Arabic numerals) are numbered according to their appearance in the amino acid sequence of the respective protein; e.g. C1 = MKNPKKKSGGFRIVN = amino acid position 1-15. Positive responses obtained with matrix pools were further tested using individual peptides to confirm a positive result.

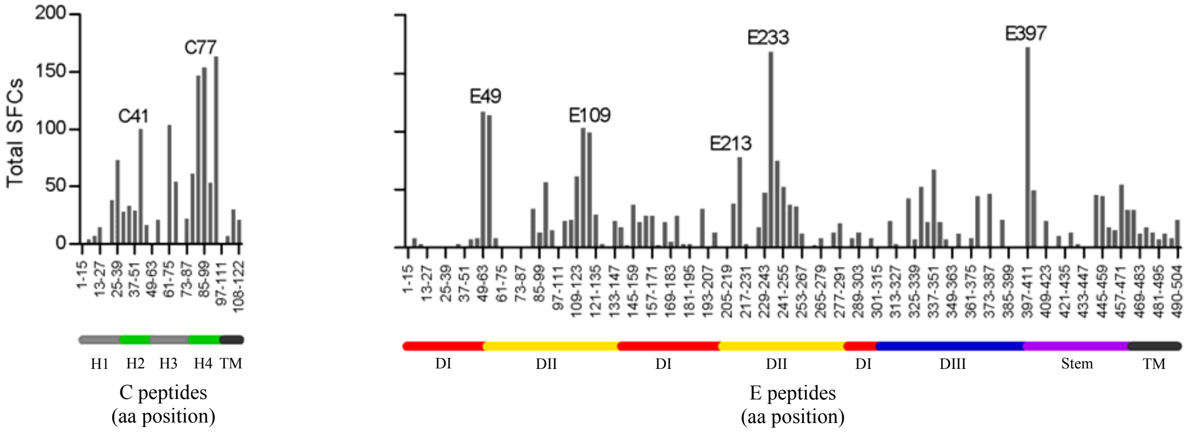

Supplementary Figure 2. Mapping of CD4 T cell responses for Zika virus C and E proteins. Total spot count of all responses to single peptides in Zika patients. Amino acid positions of peptides in C (left panel) and E (right panel) protein sequences are indicated on the x-axes. Epitope clusters are denoted by the first amino acid of the N-terminal 15-mer peptide used for single peptide testing. Positions of corresponding protein domains are shown below the x-axes as colored bars: alpha helix 1-4 (H1-H4) and the transmembrane domain (TM) of the C protein (left panel); domain I-III (DI-III), stem and transmembrane domain (TM) of the E protein (right panel). SFCs, spot forming cells.

.
